# TRIP6 a potential diagnostic marker for colorectal cancer with glycolysis and immune infiltration association

**DOI:** 10.1038/s41598-024-54670-0

**Published:** 2024-02-19

**Authors:** Xu-Sheng Liu, Yu-Xuan Chen, Hua-Bing Wan, Ya-Lan Wang, Yang-Yang Wang, Yan Gao, Li-Bing Wu, Zhi-Jun Pei

**Affiliations:** 1 Department of Nuclear Medicine, Hubei Provincial Clinical Research Center for precision Diagnosis and Treatment of liver cancer, Taihe Hospital, Hubei University of Medicine, Shiyan, 442000 Hubei China; 2 Hubei Provincial Clinical Research Center for Umbilical Cord Blood Hematopoietic Stem Cells, Taihe Hospital, Hubei University of Medicine, Shiyan, 442000 China; 3 Hubei Key Laboratory of Embryonic Stem Cell Research, Shiyan, 442000 Hubei China

**Keywords:** TRIP6, Colorectal cancer, Prognostic, Glycolysis, Immune cell infiltration, Cancer, Cancer genomics, Cancer metabolism, Tumour biomarkers

## Abstract

Thyroid hormone receptor interactor 6 (TRIP6) it is an adaptor protein belonging to the zyxin family of LIM proteins, participating in signaling events through interactions with various molecules. Despite this, TRIP6's role in colorectal cancer (CRC), particularly its correlation with glucose metabolism and immune cell infiltration, remains unclear. Through the TCGA and GEO databases, we obtained RNA sequencing data to facilitate our in-depth study and analysis of TRIP6 expression. To investigate the prognostic value of TRIP6 in CRC, we also used univariate Cox regression analysis. In addition, this study also covered a series of analyses, including clinicopathological analysis, functional enrichment analysis, glycolysis correlation analysis, immunoinfiltration analysis, immune checkpoint analysis, and angiogenesis correlation analysis, to gain a comprehensive and in-depth understanding of this biological phenomenon. It has been found that TRIP6 expression is significantly upregulated in CRC and correlates with the stage of the disease. Its overexpression portends a worse survival time. Functional enrichment analysis reveals that TRIP6 is associated with focal adhesion and glycolysis. Mechanistically, TRIP6 appears to exert its tumorigenic effect by regulating the glycolysis-related gene GPI. A higher level of expression of TRIP6 is associated with an increase in the number of iDC immune cells and a decrease in the number of Th1 immune cells. Also, TRIP6 may promote angiogenesis in tumor cells by promoting the expression of JAG2. Our study uncovers the upregulation of TRIP6 in CRC, illuminating its prognostic and diagnostic value within this context. Furthermore, we examine the relationship between TRIP6 expression levels, glycolysis, angiogenesis and immune cell infiltration. This underscores its potential as a biomarker for CRC treatment and as a therapeutic target.

## Introduction

A common malignant tumor and a major cause of death in the world, colorectal cancer is one of the most prevalent malignant tumors^1,2^. Although the diagnosis and treatment of colorectal cancer have improved significantly in recent years due to the strengthening of preventive measures and the advancement of science and technology, the prognosis is still not ideal^3,4^. Therefore, it is of great practical significance to study the deep mechanisms of the occurrence and development of colorectal cancer for formulating more effective treatment measures.

TRIP6, also known as thyroid hormone receptor interactor 6, is a novel protein which is highly consistent in human and mouse cells, suggesting it has critical physiological functions. It is an adaptor protein belonging to the zyxin family of LIM proteins, which are involved in signaling events through interactions with various molecules^5–7^. The biological function and role of TRIP6 in different cancers have been widely investigated in various clinical and biomedical studies. As a result of these studies, it has become possible to provide valuable insight into the potential role of TRIP6 in cancer progression and metastasis, a role that may be applied to the diagnosis and treatment of cancer^8–19^.

Scientists have paid close attention to the role glycolysis plays in tumor growth. Cell proliferation and survival are dependent upon glycolysis^20^. Several scholarly investigations have discovered a strong correlation between the extent of immune cell infiltration in tumor cells and the prognosis of colorectal cancer. As such, it is indicative that the progression of the disease can indeed be anticipated by quantifying the count of immune cells in proximity to the tumor cells. This insinuation arises from the fact that the infiltration of immune cells can function as a predictive criterion for determining the severity of colorectal cancer^21,22^. At the same time, studies have also found that the lactic acid produced by glycolysis and the infiltration of immune cells can also assist in the angiogenesis process of tumors^23–26^. Therefore, mining target genes related to glycolysis, angiogenesis and immune infiltrating cells has a positive role in promoting the development of new therapeutic approaches. However, there are currently no studies on the relationship between TRIP6 and CRC tumor cell glycolysis, angiogenesis, and immune cell infiltration. In-depth research on this phenomenon will help to provide new guidance for the immunotherapy of colorectal cancer.

**Figure 1:**
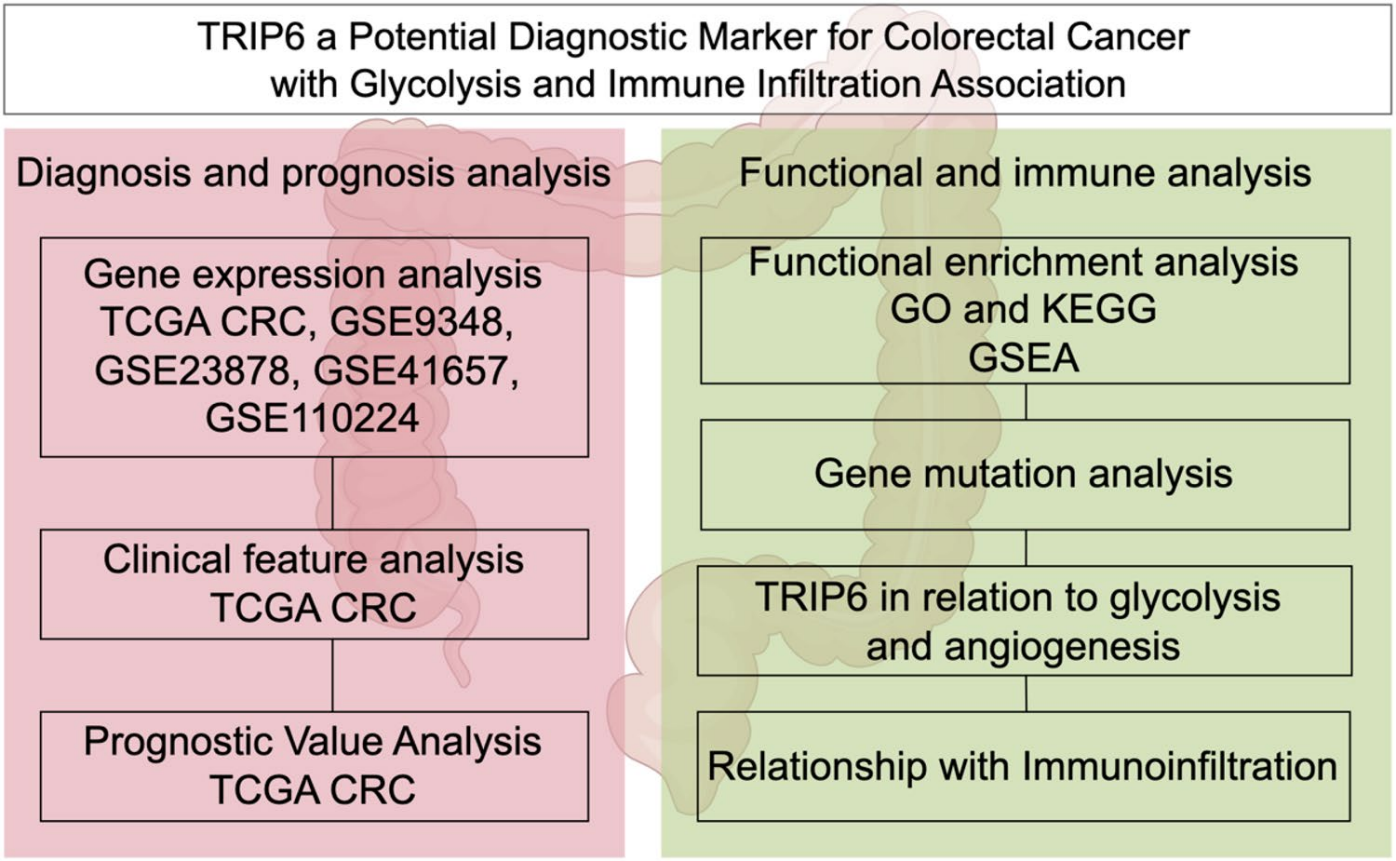
Experimental design. The figure was created by Figdraw (www.figdraw.com).

A systematic evaluation of TRIP6 function and clinical significance was conducted in this study. Our study examined mRNA expression of TRIP6 and its association with clinical prognosis using data from The Cancer Genome Atlas (TCGA) and the Gene Expression Omnibus (GEO). An analysis of Cox regression was used to determine the prognostic value of TRIP6. We also analyzed the association of genetic alterations of TRIP6 with patients with CRC. We identified co-expressed and differential genes associated with TRIP6 expression to further explore gene function. Gene ontology (GO), Kyoto Encyclopedia of Genes and Genomes (KEGG), and Genome Set Enrichment Analysis (GSEA) were used to explore the potential biological function of TRIP6. The potential association between TRIP6 and CRC glycolysis and angiogenesis was explored by analyzing the TCGA CRC and GEO datasets. As a final step, we used the ssGSEA algorithm to analyze the relationship between TRIP6 expression and immune cell infiltration rate. The research process is shown in Fig. 1.

## Materials and methods

### Public database

CRC patients' clinical information as well as gene expression profiles were obtained from the TCGA (http://portal.gdc.cancer.gov) database^27^. In addition, four datasets were collected from the GEO (http://www.ncbi.nlm.nih.gov/geo, GSE9348, GSE23878, GSE41657 and GSE110224) database^28^. As part of the preprocessing, the probes were corrected for background, normalized, and the expressions were calculated, followed by replacing the probes with gene symbols from the platforms' annotation files.

### Comparison of the TRIP6 expression level

Based on TCGA and GEO data, we measured the expression of TRIP6 gene in CRCs. The T, N, M, and pathologic stages of CRC were classified according to the National Comprehensive Cancer Network (NCCN, https://www.nccn.org/).

### Diagnostic and prognostic capability analysis

Receiver operating characteristic (ROC) curves were plotted using the TCGA CRC dataset to examine TRIP6's diagnostic significance in differentiating CRC from normal tissues. Additionally, we performed proportional hazards assumption testing and conducted survival regression analysis using the survival package to investigate the correlation between TRIP6 mRNA expression and OS (Overall Survival), DSS (Disease-Specific Survival), and PFI (Progress Free Interval), and visualized the results using the survminer and ggplot2 packages. Furthermore, we conducted univariate Cox regression analysis, including age, gender, stage, CEA expression levels, and TRIP6 expression levels, to assess the impact of each parameter on OS, DSS, and PFI.

### Functional enrichment analysis

A Pearson correlation analysis was conducted on TCGA CRC dataset to examine the association between TRIP6 expression and other genes. To visually represent all the correlation analysis results, volcano plots were generated. Additionally, chord diagrams were created to depict the top 10 genes that were positively and negatively correlated with TRIP6 expression levels. Furthermore, genes encoding proteins with a correlation coefficient (cor) > 0.3 and a *P* < 0.05 were retained as co-expressed genes of TRIP6. To further analyze the co-expressed genes, we employed the clusterProfiler package to perform gene ontology (GO) and Kyoto Encyclopedia of Genes and Genomes (KEGG) enrichment analysis. Finally, we visualized the enrichment analysis results using the ggplot2 package.

Based on TRIP6 expression levels in the TCGA CRC dataset, samples were categorized as high or low expression. On the TCGA CRC dataset, differential expression analysis was performed using the DESeq2 package. By utilizing the gene set database reference (https://www.gsea-msigdb.org/gsea/msigdb/collections.jsp, c2.cp.all.v2022.1.Hs.symbols.gmt), clusterProfiler was utilized to perform gene set enrichment analysis (GSEA) on differentially expressed genes^29^. It is considered statistically significant when data have a P value of 0.05 or less. To visualize the enrichment analysis results, the package ggplot2 was used.

### Genetic alterations of the TRIP6 gene in CRC

We investigated copy number variations and mutations in TRIP6 using the colorectal cancer (TCGA, PanCancer Atlas) dataset downloaded from the cancer genomics dataset's open-source database cBioPortal (https://www.cbioportal.org/)^30,31^. In CRC patients, we investigated the effect of genetic alterations on TRIP6 gene expression and prognosis.

### Analysis of TRIP6 in relation to glycolysis and angiogenesis

In order to study the potential relationship between TRIP6 expression and glycolysis and angiogenesis, we analyzed the TCGA CRC dataset and the GES110224 dataset. According to the data information of GES110224, we analyzed the correlation between TRIP6 expression and ^18^F-FDG metabolic parameter (standardized uptake value, SUV) in CRC samples to explore the potential connection between TRIP6 expression and glycolysis. Secondly, GSEA analysis was used to determine whether the differential genes related to TRIP6 expression are involved in glycolysis pathways, and the correlation between TRIP6 and glycolysis signatures was analyzed. Then the relationship between TRIP6 expression level and glycolysis-related and angiogenesis-related genes was further analyzed in the above two datasets. Based on the level of TRIP6 expression in the two datasets, we compared the expression levels of glycolysis-related and angiogenesis-related genes between high and low expression groups. Finally, it was displayed through Veen diagram. The list of genes associated with angiogenesis refers to previous studies^32^.

### Immune profile analysis

Based on the ssGSEA algorithm provided in the R package-GSVA^33^, the markers of 24 immune cells provided in the Immunity article were used to calculate the immune infiltration status of the corresponding cloud data^34^. For the specific 24 immune cells, please check the corresponding references. The Spearman correlation between TRIP6 expression and immune cell infiltration level was analyzed. Wilcoxon rank sum test was performed to determine the potential connection between infiltrating immune cells and TRIP6 expression groups. In an effort to provide a more nuanced understanding of the potential interaction mechanism between TRIP6 and the infiltration of immune cells, an exhaustive analysis was conducted on the correlation between TRIP6 expression and various key factors within the immune system. This comprehensive analysis incorporated the examination of immunostimulator genes, immunoinhibitor genes, as well as genes of the major histocompatibility complex (MHC). The aim was to establish a clearer picture of TRIP6's role and interplay with these different components within the immune system^35,36^.

## Results

### Expression and diagnostic value of TRIP6 in CRC

**Figure 2:**
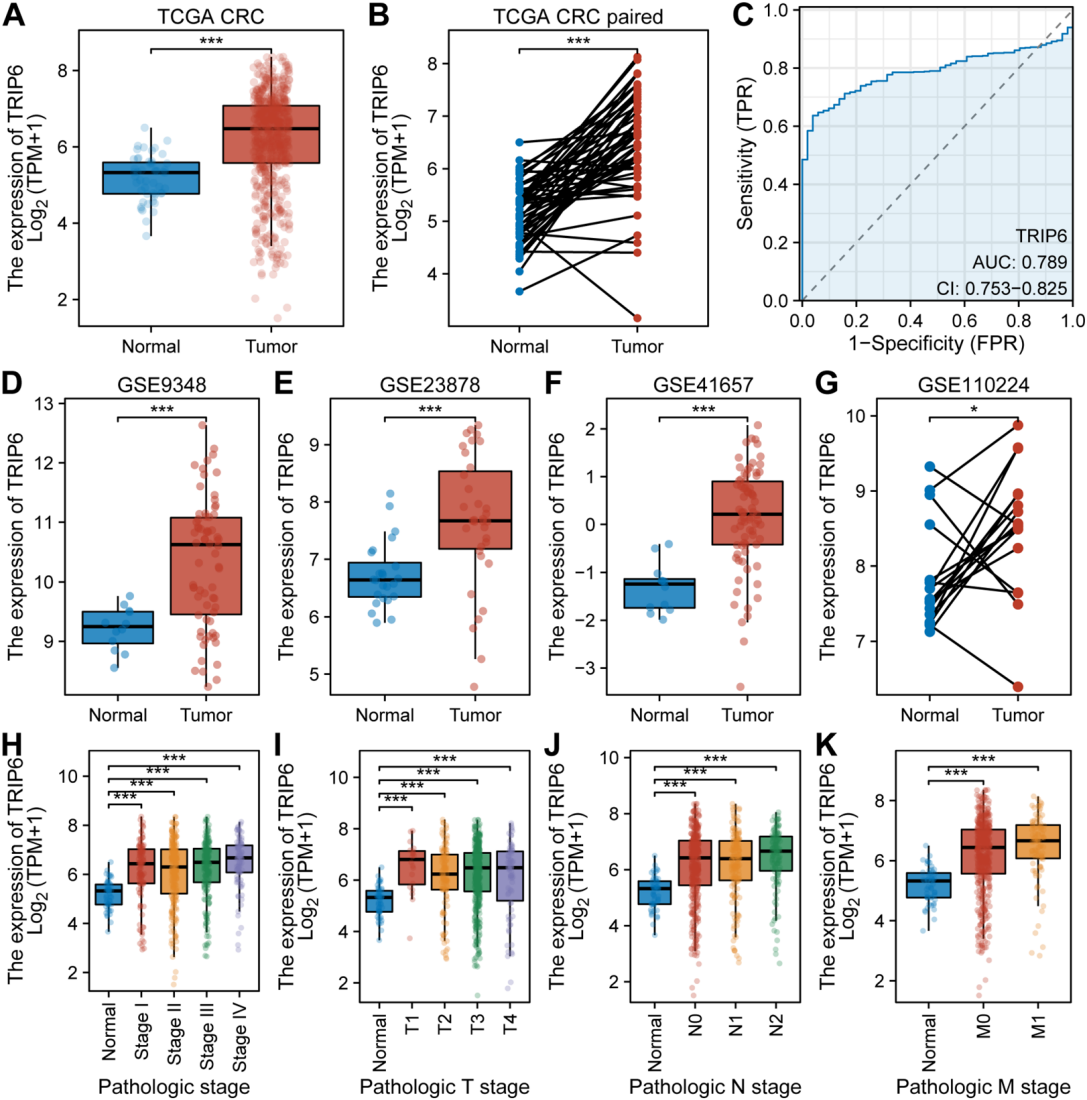
TRIP6 expression was significantly elevated and correlated with malignant clinicopathological parameters of CRC. (**A**,**B**) Data from the TCGA dataset showed that TRIP6 mRNA was up-regulated in tumor tissues compared to non-tumor tissues (*P* < 0.05). (**C**) Diagnostic ROC curve of TRIP6 in the TCGA CRC dataset. (**D**–**G**) Data from the GEO database showed that TRIP6 mRNA was up-regulated in tumor tissues compared to non-tumor tissues (*P* < 0.05). (**H**–**K**) TRIP6 expression was associated with pathologic stage, T stage, N stage, M stage (*P* < 0.05). *** *P* < 0.001, ** *P* < 0.01, * *P* < 0.05.

We evaluated the expression levels of TRIP6 in CRC tissues and non-tumor tissues using data from the TCGA database to establish a correlation between TRIP6 and CRC. No matter if non-paired samples were analyzed or paired samples were analyzed, the level of TRIP6 expression in CRC was significantly greater than in the control group (Fig. 2A,B, *P* < 0.05). We further conducted ROC curve analysis to demonstrate the diagnostic value of TRIP6. TRIP6 expression had an area under the curve (AUC) ranging from 0.753 to 0.825, which indicates a correlation between TRIP6 and CRC diagnosis (Fig. 2C). Subsequently, we analyzed the data from 4 GEO databases to further validate the expression levels of TRIP6 in CRC tissues and non-tumor tissues. The results showed that the mRNA expression level of TRIP6 in CRC was significantly increased (Fig. 2D–G). Furthermore, the expression of TRIP6 was also associated with pathological staging, T staging, N staging, and M staging (Fig. 2H–K, *P* < 0.05).

### Prognostic value of TRIP6 in CRC

**Figure 3:**
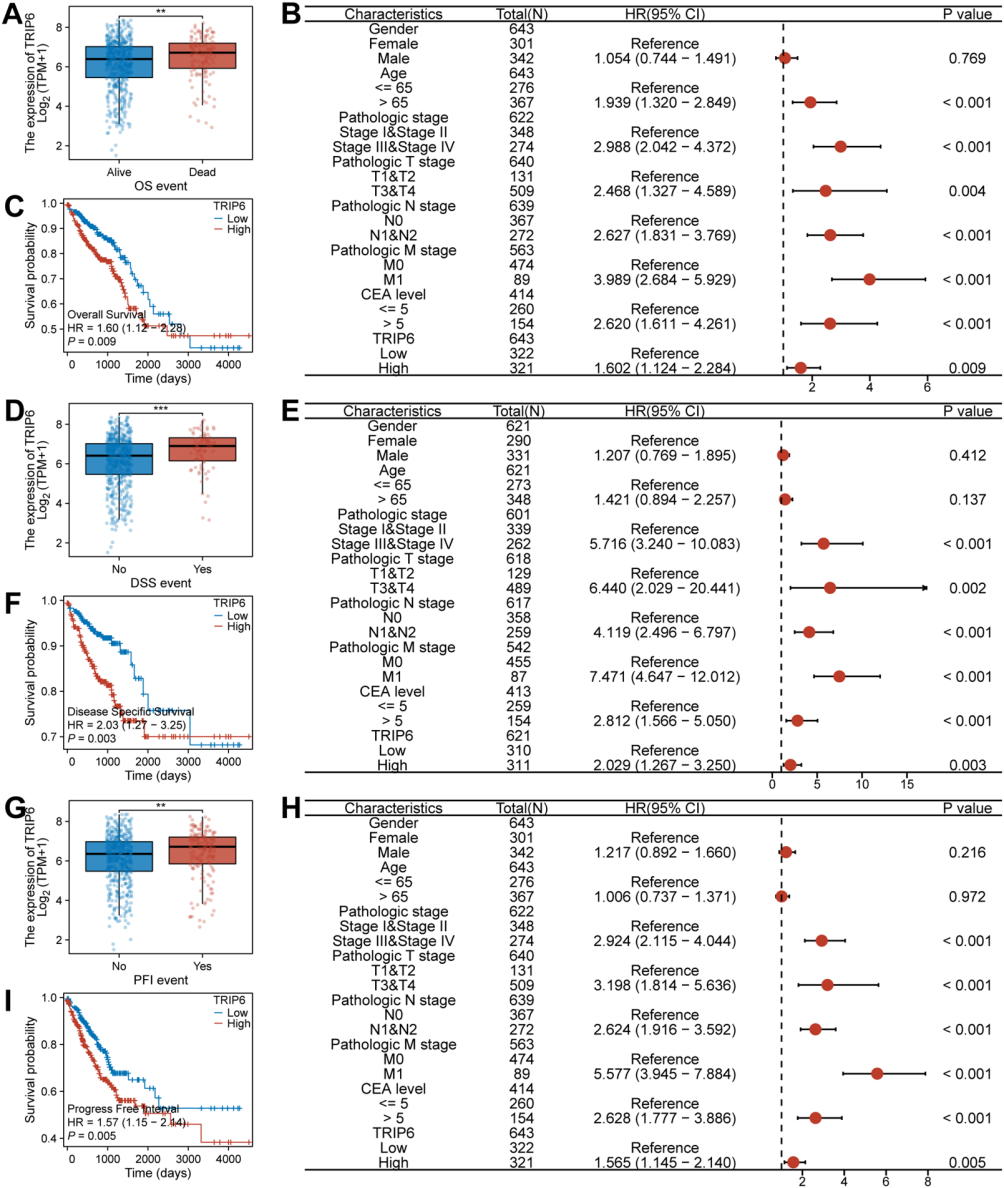
High expression of TRIP6 predicts a worse survival prognosis. (**A**,**D**,**G**) TRIP6 expression was associated with OS event, DSS event and PFI event (*P* < 0.05). (**C**,**F**,**I**) The KM plotter evaluated the prognostic value of TRIP6 expression for OS, DSS, and PFI (*P* < 0.05). (**B**,**E**,**H**) Univariate Cox regression analysis determined that pathological staging, T staging, N staging, M staging, CEA expression, and TRIP6 expression were important prognostic factors (*P* < 0.05).

We examined the differential expression of TRIP6 among different groups in relation to OS events, DSS events, and PFI events using gene expression transcripts and clinical data. The results showed that in the OS event, the expression level of TRIP6 in the death group was significantly higher than that in the survival group (Fig. 3A, *P* < 0.05). In the DSS event, the expression level of TRIP6 in the occurrence group was significantly higher than that in the non-occurrence group (Fig. 3D, *P* < 0.05). In the PFI event, the expression level of TRIP6 in the occurrence group was significantly higher than that in the non-occurrence group (Fig. 3G, *P* < 0.05). Additionally, we used KM plotter to evaluate the prognostic value of TRIP6 expression in OS, DSS, and PFI. The results showed that high expression of TRIP6 indicated a worse survival prognosis (Fig. 3C, F, I, *P* < 0.05). Univariate Cox regression analysis determined that pathological staging, T staging, N staging, M staging, CEA expression, and TRIP6 expression were important prognostic factors (Fig. 3B, E, H, *P* < 0.05).

### Correlation analysis and functional enrichment of co-expressed genes with TRIP6 in CRC

**Figure 4:**
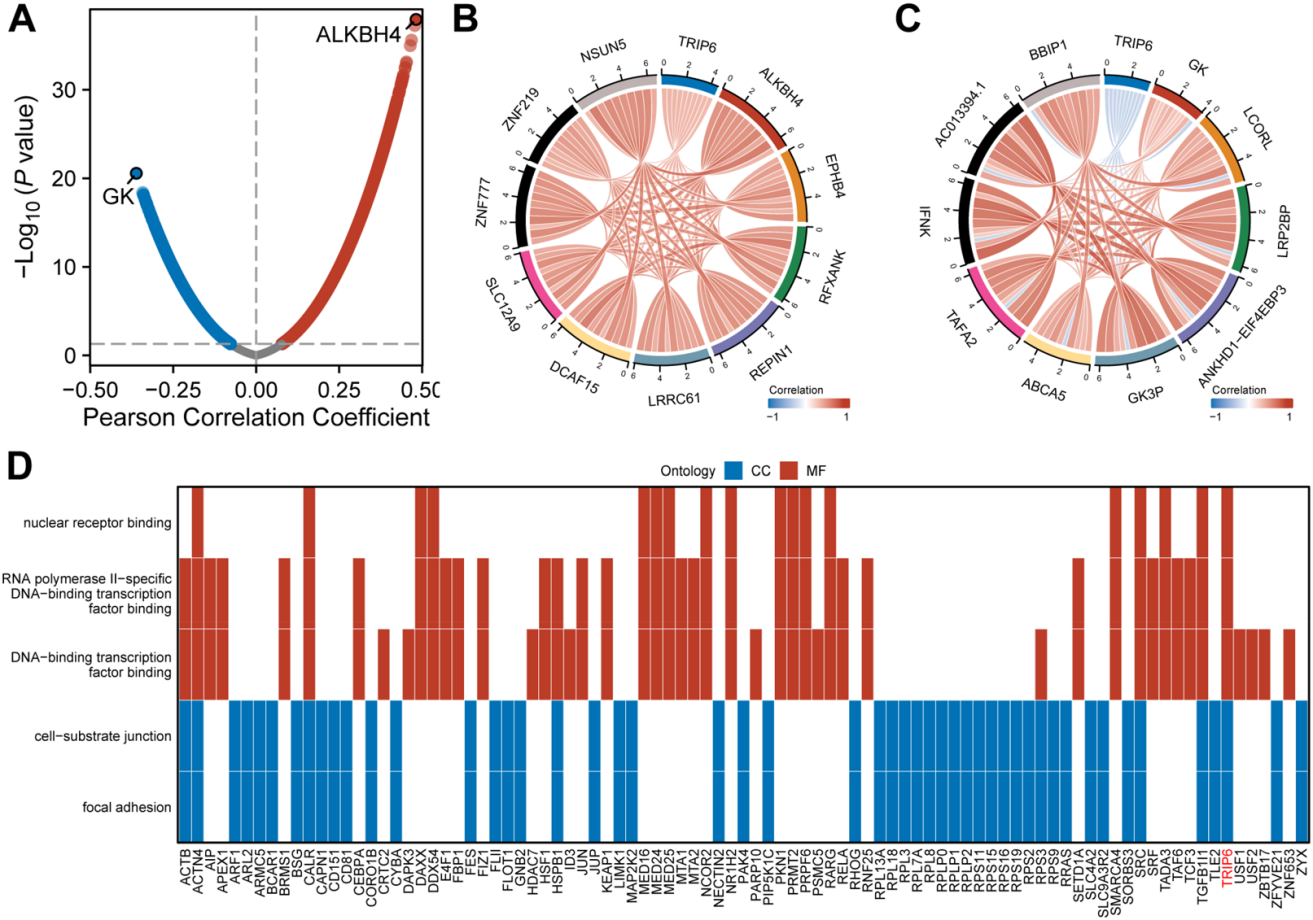
Illustrates the correlation analysis and functional enrichment of co-expressed genes with TRIP6 in colorectal cancer (CRC). (**A**) The volcano map depicts the correlation patterns of TRIP6 with other genes. (**B**) Correlation analysis is conducted between TRIP6 and the ten most positively correlated genes, while (**C**) correlation analysis is also performed between TRIP6 and the ten most negatively correlated genes. (**D**) Additionally, the diagram visually represents the potential biological pathways in which TRIP6, and its co-expressed genes may be involved in CRC.

To gain a better understanding of the biological function of TRIP6, we utilized Pearson correlation analysis to examine the correlation between TRIP6 and each gene in the TCGA CRC dataset. We found that at a significance level of *P* < 0.05 and within the range of protein-coding genes, 6573 genes showed a positive correlation with TRIP6, while 5,133 genes showed a negative correlation. Interestingly, the expression of ALKBH4 was found to have the highest positive correlation coefficient with TRIP6, while GK exhibited the highest negative correlation coefficient (Fig. 4A). In addition, a string diagram was constructed to identify the top 10 positively correlated genes with TRIP6 (Fig. 4B) and negatively correlated genes (Fig. 4C). To narrow down our investigation, we set thresholds of correlation (cor) > 0.3 and *P* < 0.05, and finally identified a total of 1109 co-expressed genes. We performed further GO and KEGG enrichment analysis of these co-expressed genes, revealing 5 biological pathways (2 cellular components and 3 molecular functions, *P* < 0.05) that included TRIP6 genes. Our results suggest that TRIP6 and co-expressed genes may be involved in focal adhesion, cell-substrate junction, DNA-binding transcription factor binding, RNA polymerase II-specific DNA-binding transcription factor binding, and nuclear receptor binding (Fig. 4D).

### Gene alterations are associated with dysregulation of TRIP6 expression in CRC patients

**Figure 5:**
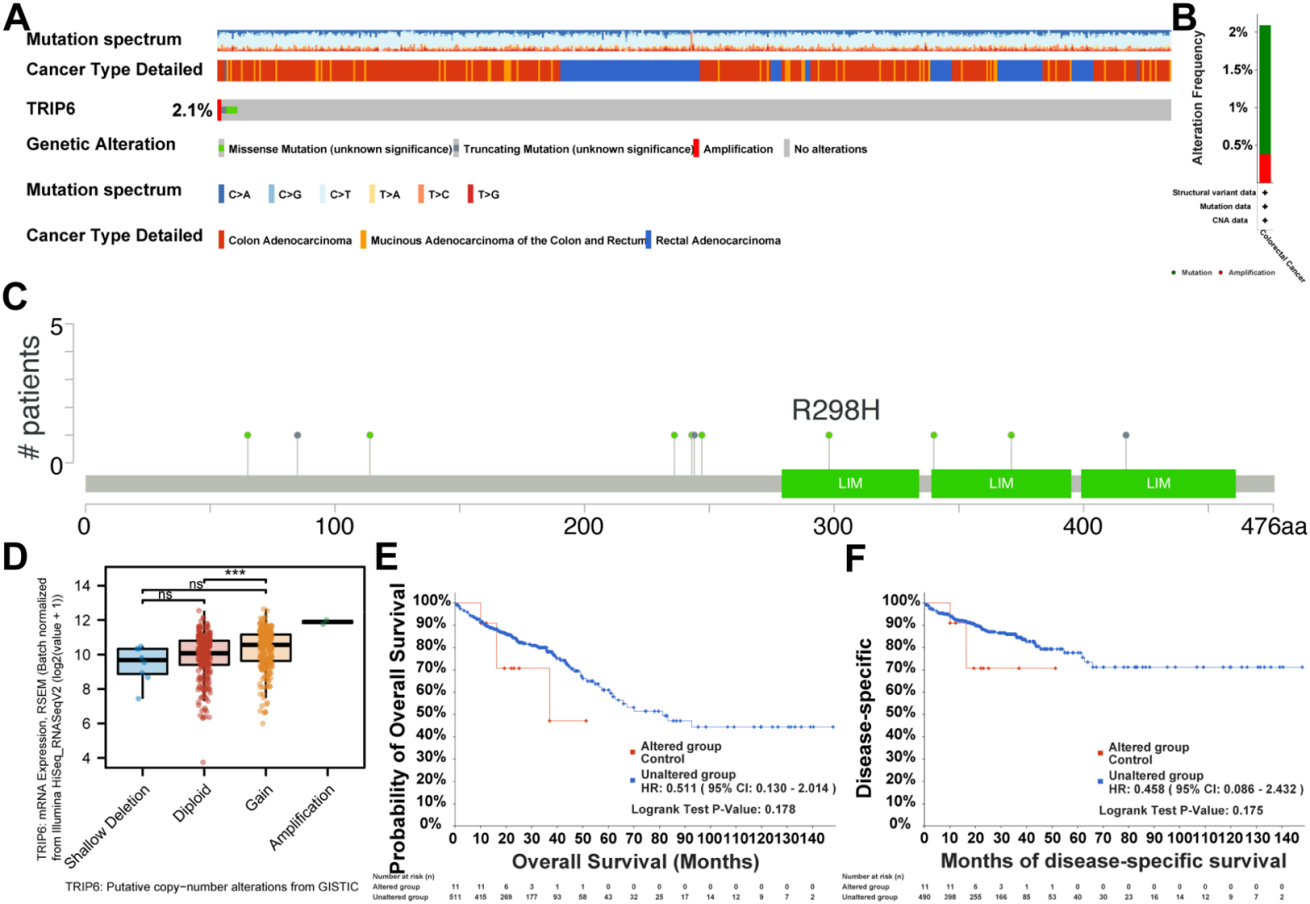
Gene alterations are associated with dysregulation of TRIP6 expression in CRC patients. (**A**, **B**) TRIP6 alterations were detected in the cBioPortal database in 2% of patients surveyed. (**C**) R298H was the most common mutation check point. (**D**) TRIP6 mRNA expression dysregulation associated with CRC copy number alterations. (**E**,**F**) There were no statistically significant differences in overall survival and disease-specific survival between patients with TRIP6 alterations and those without TRIP6 alterations.

We investigated genetic alterations in TRIP6 in 526 CRC patients in the cBioPortal database and found that alterations in TRIP6, including Mutation and Amplification, were detected in 11 (2%) of the patients who were queried (Fig. 5A,B). As shown in Fig. 5C, there were 11 mutations in the full sequence of TRIP6. In addition, “Missense” appeared to be the predominant type of genetic alteration, with R298H being the most common mutation check point. In addition, dysregulation of TRIP6 mRNA expression was associated with alterations in copy number in the cBioPortal database (Fig. 5D). In addition, survival analysis showed no statistical difference in overall survival and disease-specific survival between patients with TRIP6 alterations and those without them (Fig. 5E,F).

### Analysis of the relationship between TRIP6 and glycolysis

**Figure 6:**
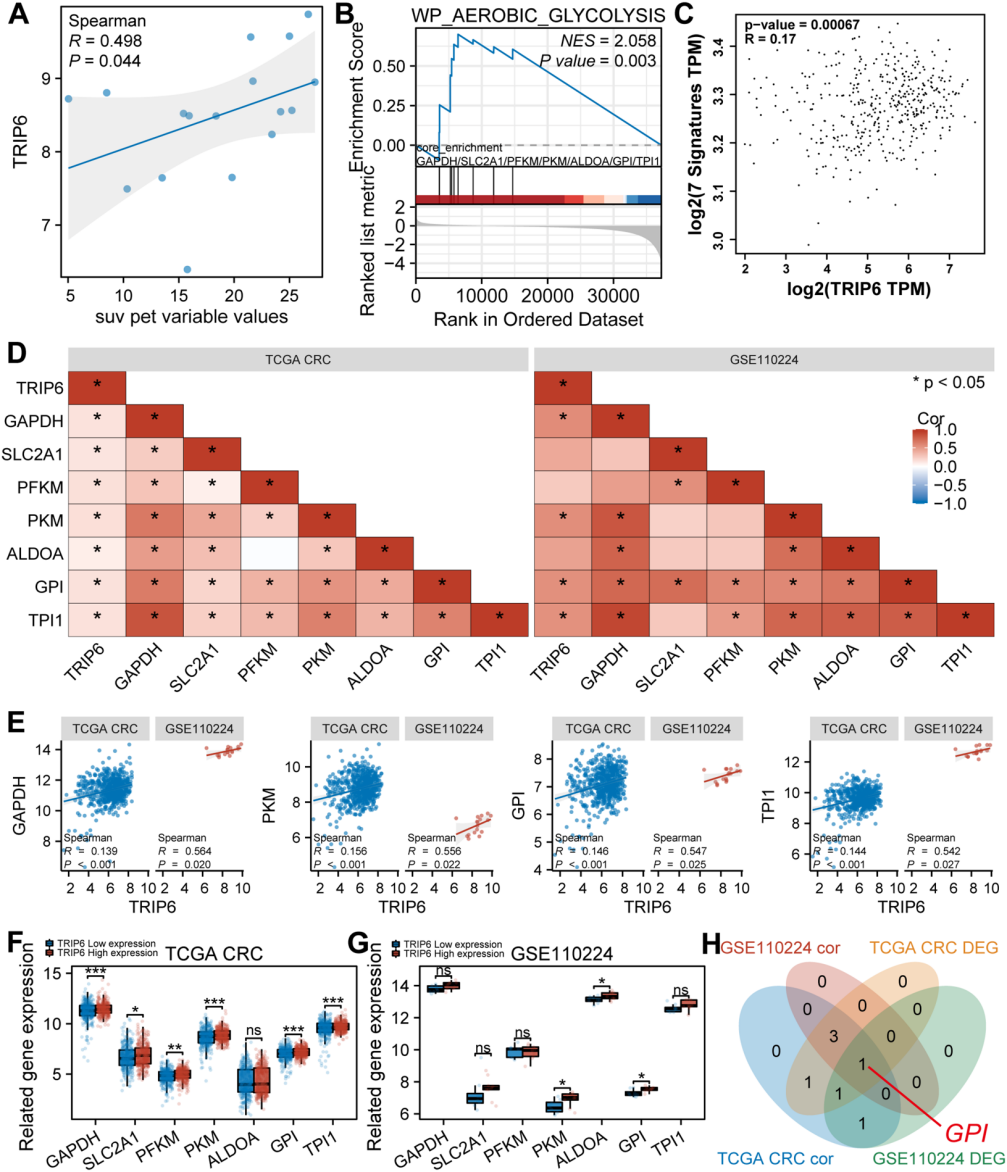
Analysis of the relationship between TRIP6 and glycolysis. (**A**) Correlation between TRIP6 expression and ^18^F-FDG metabolic parameter (SUV) in the GSE110224 dataset. (**B**) GSEA analysis revealed that differential genes related to TRIP6 expression may be involved in the glycolytic pathway. (**C**) GEPIA online tool analysis found that TRIP6 was significantly positively correlated with these seven glycolytic-related glycolytic signatures. (**D**) The relationship between TRIP6 and glycolytic-related gene expression in the TCGA CRC and GSE110224 datasets. (**E**) Glycolytic genes significantly positively correlated with TRIP6 expression. (**F**) In the TCGA CRC dataset, there were significant differences in the expression of six glycolysis-related genes between the high and low TRIP6 groups. (**G**) In the GSE110224 dataset, there were significant differences in the expression of three glycolysis-related genes between the high and low TRIP6 groups. (**H**) The Veen diagram revealed that only GPI genes were positive in the above analysis.

In order to explore the potential association between TRIP6 expression and glycolysis, we analyzed the correlation between TRIP6 expression and ^18^F-FDG metabolic parameter (SUV) in CRC samples, and the results showed that the expression level of TRIP6 was significantly positively correlated with the uptake value of SUV (Fig. 6A, *P* < 0.05). GSEA analysis found that differential genes related to TRIP6 expression may be involved in the glycolysis pathway (Fig. 6B, WP AEROBIC GLYCOLYSIS, NES = 2.058 *P* < 0.05), including 7 glycolysis-related genes, namely GAPDH, SLC2A1, PFKM, PKM, ALDOA, GPI and TPI1. TRIP6 was found to be significantly positively correlated with these 7 glycolysis-related glycolytic signatures by GEPIA online tool analysis (Fig. 6C, *P* < 0.05). Further analysis revealed that in the TCGA CRC and GSE110224 datasets, the expression level of TRIP6 was significantly positively correlated with the expression of GAPDH, PKM, GPI and TPI1, while the expression of TRIP6 was significantly positively correlated with SLC2A1, PFKM and ALDOA only in the TCGA CRC dataset (Fig. 6D, *P* < 0.05). Glycolytic genes significantly positively correlated with TRIP6 expression (Fig. 6E). Figure 6F shows that in the TCGA CRC dataset, there are significant differences in the expression of six glycolytic-related genes between high and low TRIP6 groups, namely GAPDH, SLC2A1, PFKM, PKM, GPI and TPI1. Figure 6G shows that in the GSE110224 dataset, there are three glycolytic-related genes with significant differences in expression between high and low TRIP6 groups, namely PKM, ALDOA and GPI. Veen diagram shows that only GPI genes are positive in the above analysis (Fig. 6H).

### Correlation of TRIP6 with immune cell infiltration and immune‐related genes

**Figure 7:**
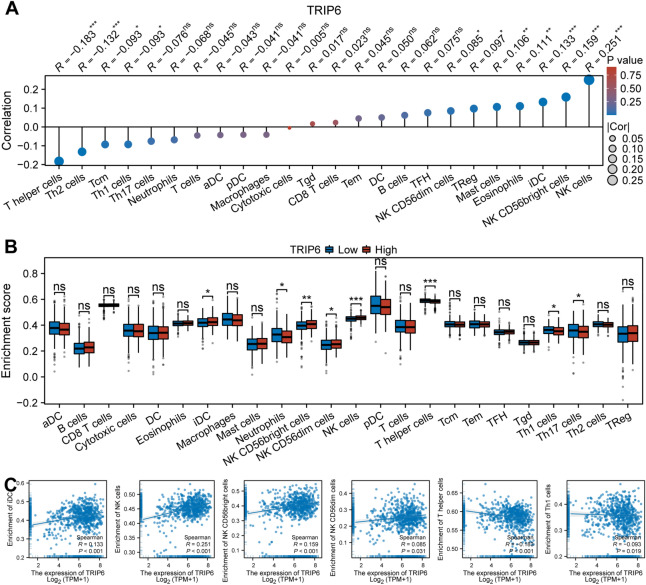
The relationship between TRIP6 expression level and immune cell infiltration. (**A**) Analysis of the relationship between TRIP6 expression level and immune cell infiltration in 24 cases. (**B**) Comparison of different immune cell infiltration levels in low TRIP6 expression group and high TRIP6 expression group. (**C**) Scatter plot showing the correlation between TRIP6 expression and the degree of infiltration of iDC, NK CD56bright cells, NK CD56dim cells, NK cells, T helper cells and Th1 cells. *, *P* < 0.05 ; * * , *P* < 0.01 ; * * * , *P* < 0.001.

**Figure 8:**
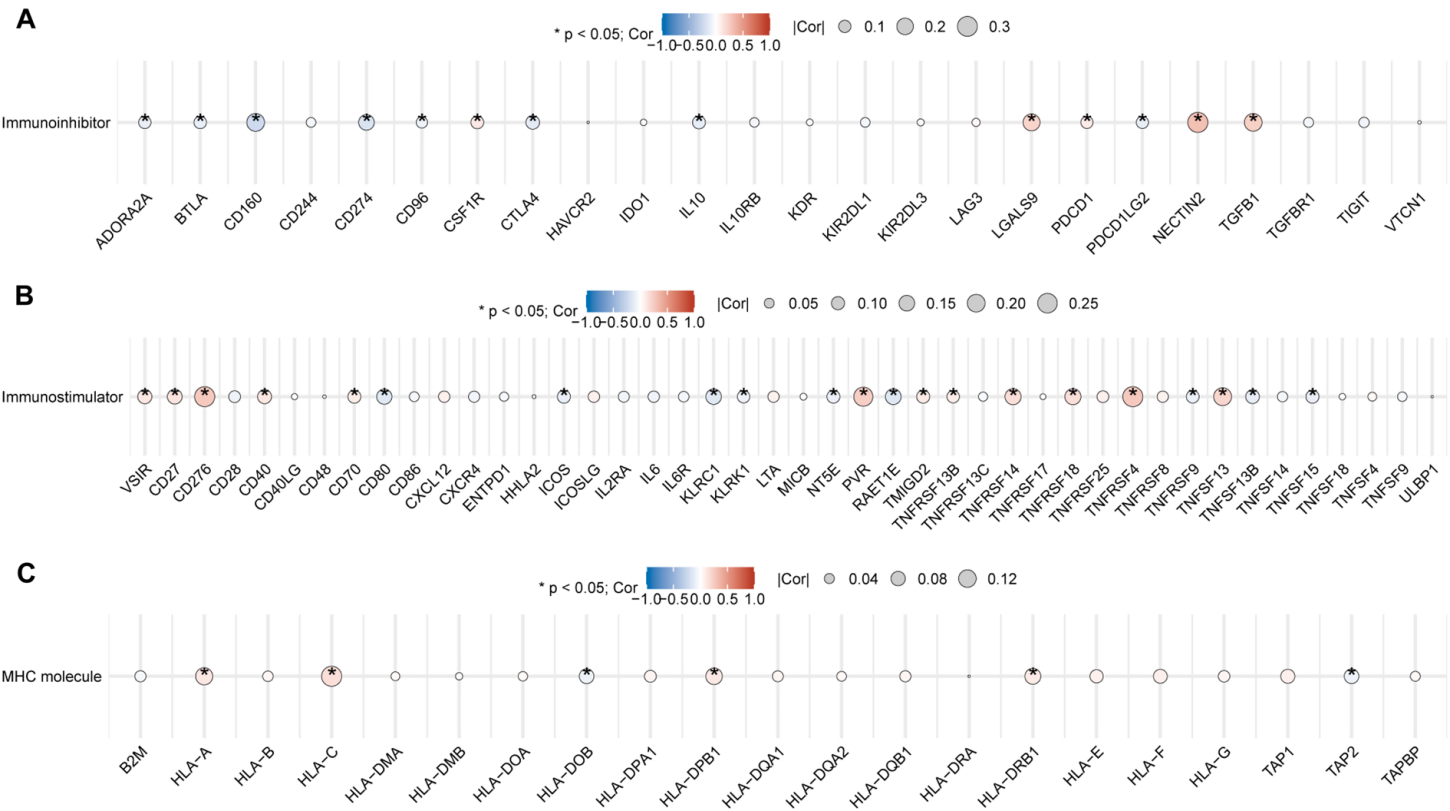
The relationship between TRIP6 expression levels and immune-related genes. (**A**–**C**) Correlation between immunostimulator genes, immunoinhibitor genes, and MHC genes and TRIP6 expression. *, *P* < 0.05; **, *P* < 0.01; ***, *P* < 0.001.

In an endeavor to unearth the potential relationship between TRIP6 expression and immune function, we conducted a meticulous investigation on 24 immune cell populations that are present within the tumor microenvironment by leveraging the ssGSEA computational method. From our rigorous analysis, it was discerned that there exists a statistically significant positive concurrence between TRIP6 expression and several immune elements, namely, Eosinophils, iDC, Mast cells, NK CD56bright cells, NK CD56dim cells, NK cells, and TReg. Conversely, a negative correlation was observed with T helper cells, Tcm, Th1 cells, and Th2 cells (Fig. 7A). To gain deeper insight into whether the TRIP6 high and low expression groups display significant disparities in terms of the tumor immune microenvironment in CRC, we conducted a comprehensive evaluation of the differentiated expression of 24 variants of immune cells. Strikingly, the outcomes of our investigation revealed a considerable rise in the presence of iDC, NK CD56bright cells, NK CD56dim cells, and NK cells in the high TRIP6 expression group. Conversely, there was a substantial reduction in Neutrophils, T helper cells, Th1 cells, and Th17 cells within the same high TRIP6 expression group. These results provide enlightening insights into the influence of TRIP6 expression on the tumor immune microenvironment in CRC (Fig. 7B). Figure 7C shows the correlation scatter plot of immune cells that concurrently fit the above analysis. In addition, we performed our results suggest a strong correlation between TRIP6 and these immune-related genes (Fig. 8A–C), which further confirms our hypothesis that TRIP6 may regulate immune cell infiltration.

### Analysis of the relationship between TRIP6 and angiogenesis

**Figure 9:**
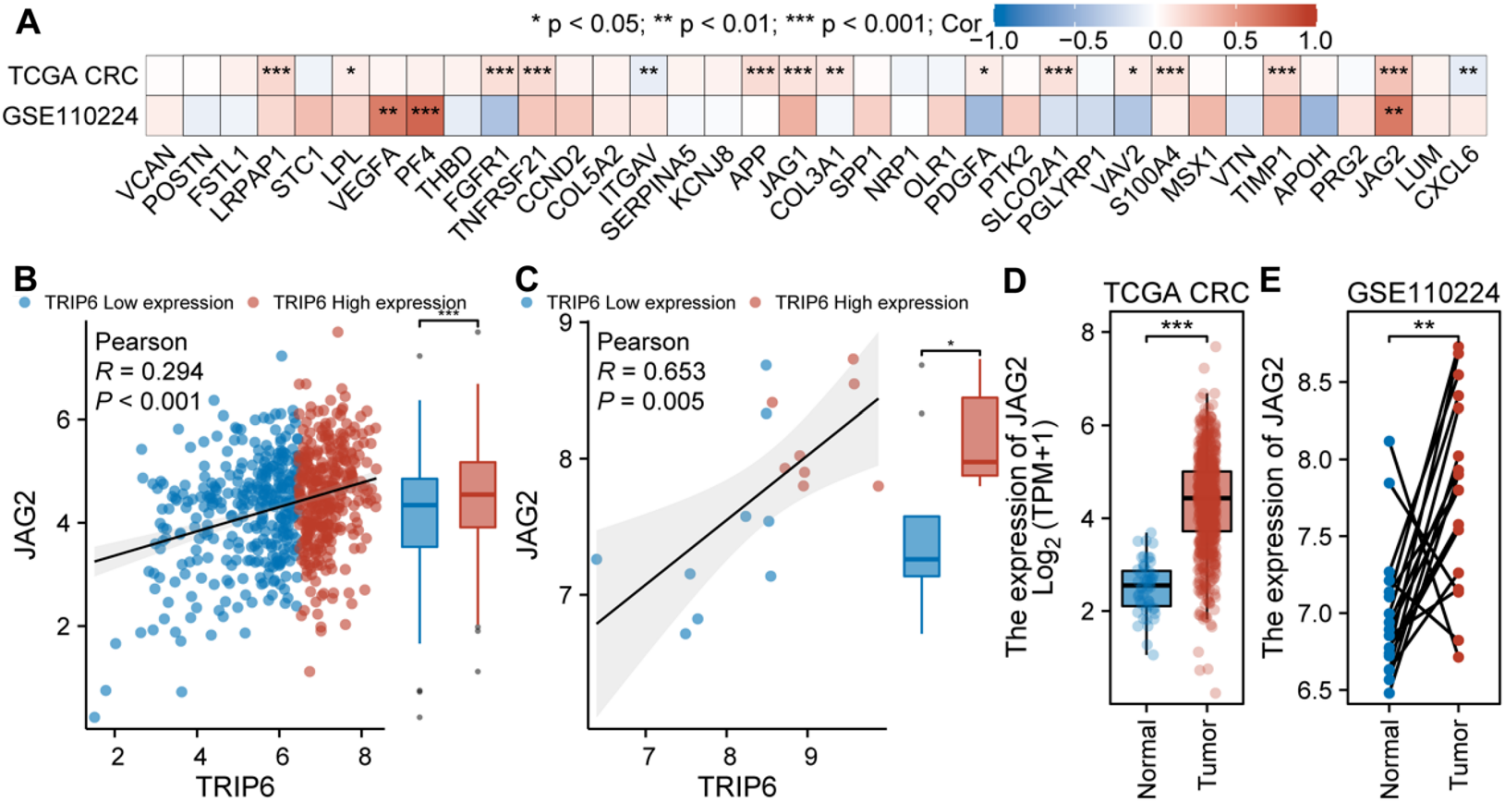
Analysis of the relationship between TRIP6 and angiogenesis. (**A**) The relationship between TRIP6 and angiogenesis-related gene expression in the TCGA CRC and GSE110224 datasets. (**B**) The correlation between TRIP6 and JAG2 in the TCGA CRC dataset, and the expression level of JAG2 in the high TRIP6 group was significantly higher than that in the low TRIP6 group. (**C**) The correlation between TRIP6 and JAG2 in the GSE110224 dataset, and the expression level of JAG2 in the high TRIP6 group was significantly higher than that in the low TRIP6 group. (**D**) In the TCGA CRC dataset, the expression of JAG2 in tumor samples was significantly higher than that in normal samples. (**E**) In the GSE110224 dataset, the expression of JAG2 in tumor samples was significantly higher than in normal samples.

To explore the potential association between TRIP6 expression and angiogenesis, we analyzed the TCGA CRC and GSE110224 datasets. It was found that in the above two datasets, the expression level of TRIP6 was significantly positively correlated with the expression of JAG2 (Fig. 9A, *P* < 0.05). In addition, in the TCGA CRC dataset, the expression of TRIP6 was significantly positively correlated with the expression of LRPAP1, LPL, FGFR1, TNFRSF21, APP, JAG1, COL3A1, PDGFA, SLCO2A1, VAV2, S100A4, and TIMP1, but significantly negatively correlated with the expression of ITGAV and CXCL6 (Fig. 9A, *P* < 0.05). In the GSE110224 dataset, the expression of TRIP6 was significantly positively correlated with the expression of VEGFA and PF4 (Fig. 9A, *P* < 0.05). Figure 9B shows the correlation between TRIP6 and JAG2 in the TCGA CRC dataset, and the expression level of JAG2 in the high TRIP6 group was significantly higher than that in the low TRIP6 group (*P* < 0.05). Figure 9C shows the correlation between TRIP6 and JAG2 in the GSE110224 dataset, and the expression level of JAG2 in the high TRIP6 group was significantly higher than that in the low TRIP6 group (*P* < 0.05). Finally, the analysis found that in the TCGA CRC and GSE110224 datasets, the expression of JAG2 in the tumor samples was significantly higher than that in the normal sample group (Fig. 9D,E, *P* < 0.05).

## Discussion

Thyroid hormone receptor interactor 6, also referred to as TRIP6, refers to a gene that is responsible for encoding the TRIP6 protein. This specific protein has an imperative role in several cellular functions including, but not limited to, cellular adhesion, cellular migration, and signal transduction processes. As a result, the TRIP6 protein is pivotal in mediating and regulating a wide array of cellular activities and processes^5–7^. Studies have shown that TRIP6 is overexpressed in different types of cancer, including breast^10^, glioma^11^, gastric^9^, liver^17,18^, and cervical^8^ cancers. Its overexpression is associated with increased tumor aggressiveness, metastasis, and poor prognosis in patients. In our study, through a query of the cBioPortal database, we found alterations in the TRIP6 gene in 11 out of 526 CRC patients (2%). Although the percentage may not seem high, even a small percentage of gene alterations may have significant biological effects in biomedical research. Particularly in the field of oncology, genetic alterations, such as mutations or amplification, have been shown to frequently lead to abnormal activation of important signaling pathways, thus promoting tumorigenesis and development^37,38^. However, in these cases, whether all of the dysregulation of TRIP6 expression is caused by genetic alterations requires further experimental studies to confirm. There may also be other regulatory mechanisms, such as non-coding RNA-based regulation, that affect the expression levels of TRIP6 mRNA by affecting its stability and translation.

Ling et al.^12^ found that miR-7 overexpression significantly inhibits the proliferation and migration of CRC cells. TRIP6 is a direct target gene of miR-7. After TRIP6 overexpression in vivo and in vitro, miR-7-mediated inhibition of CRC cell proliferation can be salvaged. In addition, TRIP6 overexpression promoted miR-7 mimic-mediated CRC cell migration and invasion. Guo et al.^13^ found that TRIP6 is overexpressed in CRC samples, and TRIP6 can promote CRC metastasis by disrupting tight junctions and activating Akt signaling through direct interaction with PARD3. The above studies confirmed that TRIP6 can promote cancer cell proliferation and survival. However, no studies have been reported on TRIP6 and tumor cell glycolysis, angiogenesis, and immune infiltration. Therefore, it is necessary to further study TRIP6 to elucidate its potential connection with glycolysis, angiogenesis, and immune infiltration in CRC, which will ultimately contribute to the advancement of cancer treatment and improve patient prognosis.

We found that the expression levels of TRIP6 in CRC samples were higher than those in the control group by bioinformatics analysis of CRC datasets from multiple different sample sources. The expression levels of TRIP6 in CRC samples of different tumor stages were higher than those in normal samples. More importantly, upregulation of TRIP6 mRNA is a valuable prognostic indicator in CRC patients. Survival analysis showed that higher TRIP6 expression was associated with shorter OS, DSS, and PFI. These results suggest that TRIP6 may be a diagnostic and prognostic biomarker for CRC.

In the co-expression analysis of TRIP6 in the TCGA CRC dataset, we observed a positive correlation between the expressions of ALKBH4 and TRIP6. Kogaki et al.^39^ have found that ALKBH4 is involved in the regulation of uridine modification and extends the role of tRNA-mediated translation control through ALKBH4. Aoki et al.^40^ found a higher level of ALKBH4 expression in lung cancer tissues relative to adjacent normal tissues. A higher proportion of ALKBH4-expressing cancer cells was observed in lung adenocarcinoma than other histological types. Multivariate logistic regression analysis indicated that ALKBH4 expression is an independent prognostic factor for RFS and OS. Additionally, we noted a negative correlation between Glycerol kinase (GK) expression and TRIP6 expression, and it has been reported that the protein encoded by this gene belongs to FGGY kinase family. This protein, as a key enzyme in regulating glycerol intake and metabolism, contributes to the co-expression of molecular chaperones for expressing biologically active human GK^41^. Studies have shown that GK5 gene silencing can induce mitochondrial damage, caspase activation, cell cycle arrest and apoptosis in PC9R cells through the SREBP1/SCD1 signaling pathway, thereby conferring lung cancer resistance to gefitinib^42^. We subsequently performed GO and KEGG enrichment analysis on the co-expressed gene set, which identified the involvement of TRIP6 co-expressed genes in the focal adhesion pathway. Studies have shown that ITGB8-AS1 can act as a competitive endogenous RNA to regulate CRC cell proliferation and tumor growth through focal adhesion signaling pathway regulation^43^. These findings suggest that TRIP6 may play a significant biological role in the occurrence and development of CRC.

Previous studies have reported that enhanced glycolytic activity in tumor cells correlates positively with ^18^F-FDG uptake. Thus, by measuring the uptake values of ^18^F-FDG (SUV), one could indirectly reflect the glycolytic activity and metabolic status of the tumor cells^44–47^. In our current study, analysis of the GSE110224 dataset revealed a positive correlation between TRIP6 expression and ^18^F-FDG metabolic parameters (SUV), suggesting that an increase in TRIP6 expression could potentially lead to significant changes in SUV levels. Concurrently, results from the GSEA and gene expression correlation analysis further confirmed that TRIP6 may influence the glycolytic capacity of tumor cells by affecting the expression of a key glycolysis gene, GPI. Prior research has reported overexpression of GPI in CRC samples, where elevated expression levels have been linked with an increase in glycolytic responses of tumor cells^48^. Yang et al.^49^ found that Circ-CTNNB1 is highly expressed in osteosarcoma tissue, and circus-CTNNB1 interacts with RBM15 to promote the expression of HK2, GPI, and PGK1 to promote glycolysis and activate osteosarcoma progression. This further elucidates that TRIP6 might influence the glycolytic capacity of CRC cells through its effect on GPI expression, though further longitudinal studies are required to validate these findings and to ascertain their direct impact on patient prognosis and survival rates. Such findings could greatly influence our understanding of CRC progression and its metabolic variations, leading to improved targeted treatments. However, a more detailed investigation involving the integration of functional genomics and metabolomics into the molecular mechanisms would be crucial in determining the definitive role of TRIP6 in glycolysis and CRC metabolism.

Interestingly, previous studies have found that the expression of tumor glycolysis-related genes is inversely associated with immune-related genes in multiple solid tumor types. High tumor glycolysis is associated with low immune infiltration and poor prognosis^50^. A plethora of empirical evidence robustly substantiates the conjecture that the intricacy and variety inherent in the tumor microenvironment's immune milieu possesses the capacity to significantly impact both tumorigenesis and metastasis. The diverse array of immune cells and molecules present within this unique ecological niche in the body are extremely interactive and their interactions can have profound influences on how tumor cells evolve and spread, thus determining the course of disease progression^51,52^. Through an in-depth analysis of the penetration of varying types of immune cells coupled with an examination of the expression of a multitude of immune-associated genes within the oncological tissue, the omnipresence of immune cells existing within a tumor can be investigated as a key constituent of the tumor's microenvironment^36,53–57^. Our analysis identified enrichment of iDC, NK cells, NK CD56bright cells, and NK CD56dim cells in the high expression group of TRIP6, which showed a positive correlation with TRIP6 expression. T helper cells and Th1 cells were enriched in the low expression group of TRIP6 and showed a negative correlation with TRIP6 expression. Previous studies have indicated that rapidly growing “angiogenic” tumors are more susceptible to iDC infiltration compared to non-angiogenic tumors, and angiogenesis depends on the presence of iDC, which enhances tumor growth^25^. However, adaptive immune cells such as CD8+ T cells and Th1 cells can secrete IFN-γ, a potent cytokine that inhibits angiogenesis and induces normalization of the TME vasculature, thereby inhibiting tumor vascular survival^58^. We also found a significant positive correlation between TRIP6 and the expression level of the angiogenesis key gene JAG2 in both datasets. He et al. found that JAG2 was significantly higher in CRC samples than in control samples and could significantly promote CRC aggressiveness^59^. Overall, these findings suggest that an increase in immune cells promoting tumor angiogenesis and a decrease in immune cells inhibiting tumor angiogenesis in the TRIP6 high expression group exacerbate the progression of cancer, leading to unfavorable tumor prognosis. Furthermore, the correlation between TRIP6 and immunostimulator genes, immunoinhibitor genes, and MHC genes further confirms its close relationship with tumor immunity.

Despite our research providing robust support for the theoretical potential of TRIP6 and its possible contributions to the initiation and progression of CRC, our study is still faced with several constraints that must be addressed. Firstly, we mainly utilized bioinformatic analysis methods leveraging public databases like TCGA and GEO to probe into the functional role of TRIP6 in CRC. However, further in vivo and in vitro experimental research is required to verify and elucidate its mode of operation in CRC. Secondly, we analyzed the prognostic value of TRIP6 using the TCGA dataset. In the future, it's necessary to further verify this point with a larger number of clinical samples to optimize error elimination. Despite these constraints, our study reveals the core role of TRIP6 in CRC, providing valuable insights for the formulation of novel therapeutic strategies for CRC patients.

## Conclusion

In this study, we conducted a comprehensive systematic analysis of the expression characteristics, prognostic, and diagnostic value of TRIP6 in the development and origin of CRC, as well as its potential mechanisms. Our findings open new perspectives for future CRC research, which may aid healthcare professionals in more precisely anticipating the prognosis of CRC patients and provide rational references in their treatment decision-making process. Significantly, the pronounced correlation exhibited between TRIP6, and glycolysis-related genes, and angiogenesis-related genes, and immune cell infiltration and immune checkpoint genes suggests that it may serve as a potential target for tumor immunotherapy.

## Data Availability

The datasets generated during and/or analysed during the current study are available from the corresponding author on reasonable request.
